# Burden and risk factors for antenatal depression and its effect on preterm birth in South Asia: A population-based cohort study

**DOI:** 10.1371/journal.pone.0263091

**Published:** 2022-02-07

**Authors:** Rasheda Khanam, Jennifer Applegate, Imran Nisar, Arup Dutta, Sayedur Rahman, Ambreen Nizar, Said Mohammed Ali, Nabidul Haque Chowdhury, Farzana Begum, Usha Dhingra, Fahmida Tofail, Usma Mehmood, Saikat Deb, Salahuddin Ahmed, Sajid Muhammad, Sayan Das, Saifuddin Ahmed, Harshita Mittal, Nicole Minckas, Sachiyo Yoshida, Rajiv Bahl, Fyezah Jehan, Sunil Sazawal, Abdullah H. Baqui

**Affiliations:** 1 Department of International Health, Johns Hopkins Bloomberg School of Public Health, Baltimore, Maryland, United States of America; 2 Aga Khan University, Department of Paediatrics and Child Health, Karachi, Sindh, Pakistan; 3 Center for Public Health Kinetics, Global Division, New Delhi, India; 4 Projahnmo Research Foundation, Abanti, Banani, Dhaka, Bangladesh; 5 Public Health Laboratory-IDC, Chake Chake, Pemba, Tanzania; 6 International Centre for Diarrhoeal Disease Research, Bangladesh, Dhaka, Bangladesh; 7 Department of Population, Family and Reproductive Health, Johns Hopkins Bloomberg School of Public Health, Baltimore, Maryland, United States of America; 8 World Health Organization (MCA/MRD), Geneva, Switzerland; Texas A&M University College Station, UNITED STATES

## Abstract

**Introduction:**

Women experience high rates of depression, particularly during pregnancy and the postpartum periods. Using population-based data from Bangladesh and Pakistan, we estimated the burden of antenatal depression, its risk factors, and its effect on preterm birth.

**Methods:**

The study uses the following data: maternal depression measured between 24 and 28 weeks of gestation using the 9–question Patient Health Questionnaire (PHQ-9); data on pregnancy including an ultrasound before 19 weeks of gestation; data on pregnancy outcomes; and data on woman’s age, education, parity, weight, height, history of previous illness, prior miscarriage, stillbirth, husband’s education, and household socioeconomic data collected during early pregnancy. Using PHQ-9 cutoff score of ≥12, women were categorized into none to mild depression or moderate to moderately severe depression. Using ultrasound data, preterm birth was defined as babies born <37 weeks of gestation. To identify risk ratios (RR) for antenatal depression, unadjusted and adjusted RR and 95% confidence intervals (CI) were calculated using log- binomial model. Log-binomial models were also used for determining the effect of antenatal depression on preterm birth adjusting for potential confounders. Data were analyzed using Stata version 16 (StataCorp LP).

**Results:**

About 6% of the women reported moderate to moderately severe depressive symptoms during the antenatal period. A parity of ≥2 and the highest household wealth status were associated with an increased risk of depression. The overall incidence of preterm birth was 13.4%. Maternal antenatal depression was significantly associated with the risk of preterm birth (ARR, 95% CI: 1.34, 1.02–1.74).

**Conclusion:**

The increased risk of preterm birth in women with antenatal depression in conjunction with other significant risk factors suggests that depression likely occurs within a constellation of other risk factors. Thus, to effectively address the burden of preterm birth, programs require developing and providing integrated care addressing multiple risk factors.

## Introduction

Depression is a common psychological disorder that affects people in all communities around the world and significantly contributes to the global burden of disease. In 2017, the World Health Organization (WHO) estimated that more than 300 million people worldwide suffer from depression [1]. The WHO suggests that depression is the leading cause of disability, as measured by Years Lived with Disability (YLDs), and the fourth leading cause of the global burden of disease [1, 2]. Both the WHO and the United States’ Center for Disease Control (CDC) estimated that depression is more common among women than men. According to the CDC, between 2013 and 2016, 10.4 percent of all women globally were suffering from depression, compared to 5.5 percent of all men. Thus, an estimated one out of ten women enter pregnancy with depression [3]. Furthermore, the risk of depression is estimated to double for women during pregnancy [4, 5], and the prevalence of depression in pregnant women has risen in recent years, which can have adverse effects for both the mother and infant [6–11]. Unfortunately, antenatal depression often remains unrecognized, particularly in low- and middle-income countries (LMIC) because of other competing priorities [8].

Depressive disorders in pregnancy are associated with adverse pregnancy and perinatal outcomes, particularly preterm birth (birth <37 completed weeks of gestation) [12–15]. Preterm birth is a major global public health problem because of its high incidence, associated high morbidity and mortality in neonates, and potential for long-term disability. Each year about 15 million babies are born preterm, accounting for 11·1 percent of all live births worldwide [16]. The incidence ranges from about 5 percent in many European countries to 18 percent in some South Asian and sub-Saharan African countries—where more than 60 percent of all preterm births occur [17]. High-income countries are also affected, with the USA being one of the top ten countries with the largest numbers of preterm births [17]. Globally, preterm birth and associated complications are the leading cause of neonatal deaths (35 percent of the world’s 2.5 million annual deaths) and the second most frequent cause (15.4 percent) of all death in children less than 5 years of age [17]. Surviving preterm infants may experience significant morbidities such as bronchopulmonary dysplasia, high-grade intraventricular hemorrhage, retinopathy of prematurity, and chronic lung disease [18–21]. Preterm births are also associated with a greater risk of lifetime disability, including reduced cognitive function and visual and hearing impairment [22], as well as greater risk of future health conditions such as type 2 diabetes, breast cancer, and cardiovascular disease [23–27]. Furthermore, the economic burden and the lifetime costs to the caregiver of a child born preterm are substantial [28].

Studies examining the association of antenatal depression with preterm birth have shown mixed results [29–31]. To date, few studies have been conducted in LMIC where there is a high burden of antenatal depression, and the majority of the preterm births occur. Due to the paucity of evidence on the burden and risk factors for antenatal depression in LMIC, and its potential association with preterm birth, policy decisions regarding routine screening and management of antenatal depression have not been feasible. Using population-based data collected from well-characterized cohorts of pregnant women in two South Asian countries—Bangladesh and Pakistan—this study estimates the prevalence of antenatal depression, examines risk factors for antenatal depression and measures the effects of antenatal depression on preterm birth.

## Materials and methods

### Study design and population

This study uses data from a population-based, cohort study designed to identify biomarkers and other risk factors of adverse pregnancy and perinatal outcomes by collecting detailed socio-economic, demographic, clinical, epidemiological, and biological data including biospecimens. The study known as the Alliance for Maternal and Newborn Health Improvement (AMANHI) enrolled 5,500 pregnant women between August 2014 and December 2018 from two South Asian countries, Bangladesh, and Pakistan. Bangladesh populations were predominantly rural with a high burden of preterm birth, residing in two sub-districts (Zakiganj and Kanaighat) of the Sylhet district located in the northeastern part of the country. The Pakistan population also had a high burden of preterm birth but resided in a peri-urban area of Karachi. The detailed methods of the AMANHI study have been published previously [32].

### Data collection

Briefly, the study sites used standardized procedures to collect detailed data during pre-pregnancy, pregnancy, delivery, and the postpartum period. Trained community health workers (CHWs) with a minimum of 10^th^-grade education, made home visits in pre-defined surveillance populations every two to three months to identify pregnancies, births, deaths, and migration in and out of the study area. The reported pregnancies were confirmed using a pregnancy test. After obtaining written informed consent, trained ultrasonologists performed an ultrasound before 20 weeks of gestation to measure gestational age. The sites used standardized questionnaires to collect detailed socioeconomic, clinical, phenotypic, and epidemiological data from the pregnant woman and her household. In addition to the routine surveillance visits, the CHWs conducted four home visits during pregnancy: before 19 weeks, 24–28 weeks, 32–36 weeks, and 38–40 weeks of gestation. The CHWs also conducted two home visits during the postpartum period: one between one and six days and the second one between 42–60 days after delivery. During the baseline home visit before 19 weeks of gestation, the CHWs collected data on the woman’s age, education, husband’s education, household socioeconomic data, history of illness, and history of prior miscarriage, stillbirth, or child loss. The data on reported maternal morbidity during the index pregnancy, anthropometry, other risk factors and exposures, and maternal depressive symptoms were collected during all three subsequent pregnancy visits. In addition, the CHWs measured blood pressure using a digital sphygmomanometer (Microlife®), proteinuria using a dipstick (Uristix®), and hemoglobin using HaemoCue. In the first postpartum home visit within 3 days of delivery, data on date and place of delivery, survival status of the fetus, and anthropometry of all live-born babies were collected. After 42 days of delivery, fieldworkers visited families to collect data on the survival status of the women and other relevant epidemiological data. Data on maternal depressive symptoms were collected by the CHWs using the 9–question Patient Health Questionnaire (PHQ-9). The CHWs received five days of training to ensure their understanding of the questionnaires and the procedures for administering the questionnaires, to reduce interviewer bias, and to minimize potential ethical concerns.

### Data quality assurance

The study manager and field supervisors monitored the data collection process. Completed questionnaires were carefully checked for completeness and data consistency by the field supervisors and the study manager. Data forms that were found to be incomplete or contained inconsistent data, during data entry and data validation, were returned to the field for checking and correction.

### Measurements

#### Outcome variables

We have used data on maternal depressive symptoms collected during the antenatal home visit conducted between 24–28 weeks of gestation. The PHQ-9 comprised of nine questions to assess the presence of depressive symptoms in the preceding two weeks. Each question is scored on a 4-point scale, ranging from 0 (not at all) to 3 (nearly every day), for a total score ranging from 0 to 27. The scores are interpreted as follows: a score of 1–4 is considered as having minimal depression, 5–9 as mild depression, 10–14 as moderate depression, 15–19 as moderately severe depression, and 20–27 as severe depression. Thus, increasing scores indicate an increased severity of depression [33]. We estimated the prevalence of depression using cut-offs of ≥8, ≥9, ≥10, ≥11 and ≥12 as these cut-offs were found to have acceptable diagnostic property in validation studies [34, 35]. In our dataset, the cut-off of ≥12 provided the highest combined sensitivity and specificity in predicting preterm birth. Also, ≥12 was recommended as the optimal cut-off in a recent validation study by Kendrick et al (2009) against more detailed diagnostic evaluations [35]. Several authors indicated that using a more conservative PHQ-9 cutoff would increase the accuracy of predicting major depressive symptoms [34, 36]. Using the cutoff of ≥12, we categorized maternal antenatal depression into two categories: none or mild depression (PHQ-9<12) and moderate to moderately severe depression (PHQ-9 ≥12). The main outcome variable, preterm birth was defined as babies born less than 37 weeks of gestation using ultrasound data collected before 19 weeks of gestation.

#### Other covariates or confounders

Maternal age was categorized into <19, 20–29 and ≥ 30 years. Parity was categorized as nulliparous, 1 child, 2–3 children, and >3 children. Maternal education was categorized into none, primary and secondary or above. Body mass index (BMI) was defined as the ratio of weight in kilograms to height in meters squared (weight in kg/height in m^2^). Using BMI, women were classified as: normal (BMI 18.5–24.9 kg/m^2^), underweight (<18.5 kg/m^2^), and overweight or obese (≥ 25.0 kg/m^2^). The consumption of tobacco (mostly chewing) by women was classified into yes or no categories. Self-reported maternal hypertension and diabetes were classified into yes and no categories. History of prior miscarriage or prior stillbirth were combined to create a variable with four categories: not applicable for nulliparous women, no prior miscarriage or stillbirth, prior miscarriage and prior stillbirth. We created household wealth scores based on housing materials and household possessions using principal component analysis. The wealth scores were used to categorize the households into wealth quintiles.

### Data analysis

We compared selected baseline characteristics of the two study sites by calculating frequency distributions of categorical variables and the mean and corresponding 95% Confidence Intervals (CI) for continuous variables (e.g., maternal age, education, BMI, and parity). We calculated prevalence of antenatal depression using various cutoffs for each of the study site. To identify risk factors for antenatal depression, we examined unadjusted associations of maternal depression (PHQ-9 ≥12) with potential risk factors (maternal age, parity, maternal education, BMI, tobacco use, self-reported hypertension, self-reported diabetes, history of stillbirth, history of miscarriage, paternal education, household wealth, and study site) using Pearson’s chi-squared test for independence. We calculated unadjusted risk ratios and 95% confidence intervals for each of the potential risk factors of maternal depression using the log binomial model. We then calculated the adjusted risk ratios and 95% confidence intervals of maternal depression using backward stepwise regression, which is a stepwise regression approach that begins with a full model and gradually eliminates variables from the regression model to identify a reduced model (p < 0.2) that best explains the data. Log-binomial and stepwise regression were also used for determining the risk ratios and 95% confidence intervals of preterm birth in women with antenatal depression and other potential risk-factors or confounders. Our *a prior* power calculation suggested power of 0.816 based on n = 4366 and preterm rate of about 13%, depression level of 10.4% (CDC) and expected effect-size of about 40%. Quantitative data were analyzed using Stata version 16 (StataCorp LP).

### Ethical approval

We obtained approval from the following ethics committees: International Centre for Diarrhoeal Disease Research Bangladesh (icddr,b) in Bangladesh (PR 12073, 23 March 2014); Aga Khan University in Pakistan (AKU Ethics Review Committee (OMB No. 0990–0279), 4359- Ped-ERC-16 and 2790-Ped-ERC-13); Zanzibar Medical Research and Ethics Committee in Tanzania (ZAMREC/0002/October/013; 25 March 2014); and WHO Institutional Review Board (IRB) (RPC532; 22 July 2014). The Bangladesh protocol was also reviewed and approved by the IRB of Johns Hopkins Bloomberg School of Public Health, USA (IRB No: 00004508; October 2017, 2013).

## Results

**Fig 1:**
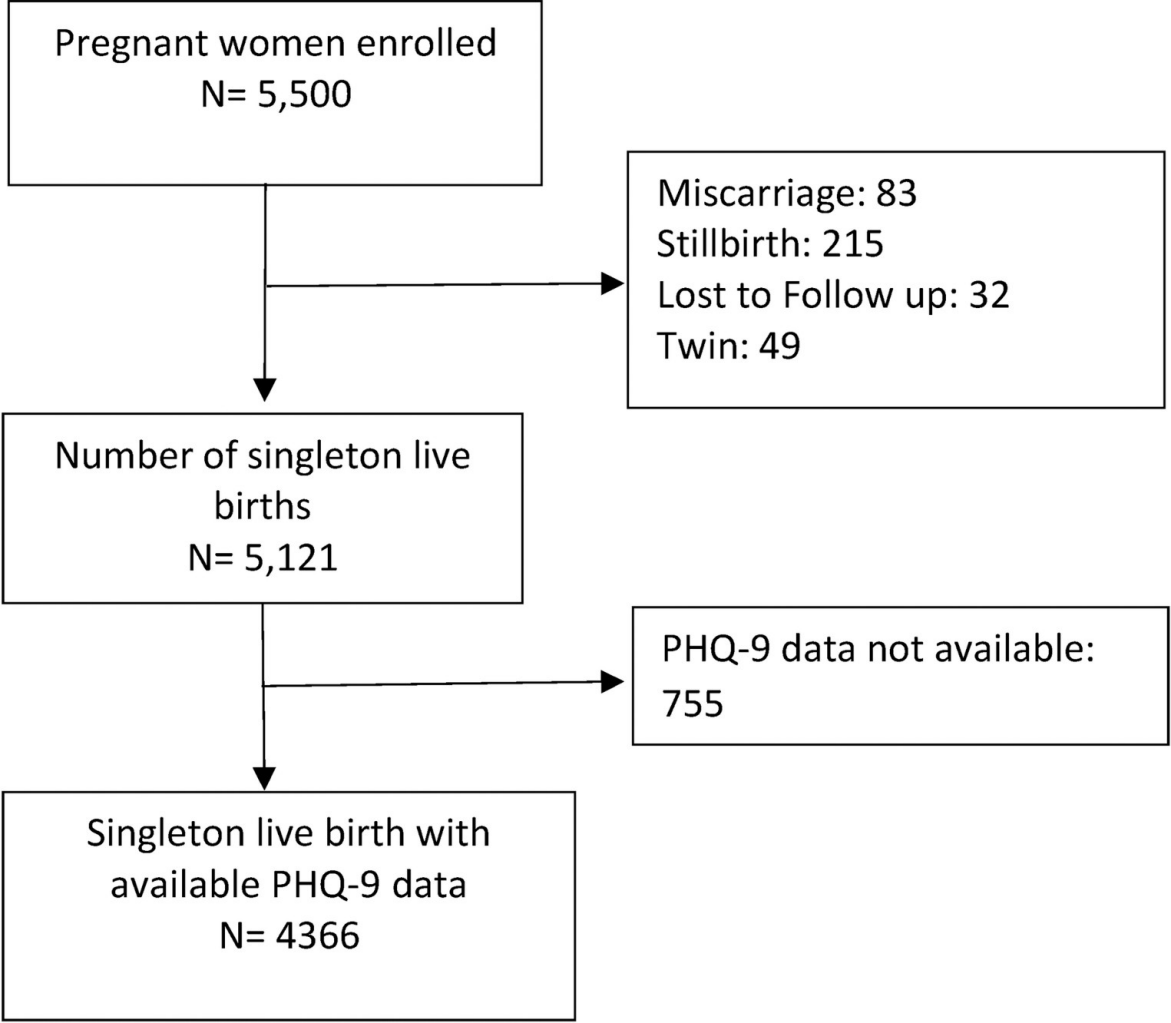
Study flowchart.

In Bangladesh and Pakistan, a total of 5,500 women were enrolled between 8–19 weeks of gestation and followed up to 42 days post-partum. Data on pregnancy outcomes and maternal depressive symptoms were available for 4,366 pregnant women [Bangladesh (N = 2,577), Pakistan (N = 1,789)] delivering a live born, singleton baby (Fig 1).

Compared to women in the Pakistan cohort, women in the Bangladesh cohort were younger (mean age in years ± 95% CI: 23.4, 23.3–23.6 vs. 26.6, 26.3–26.8), had fewer children (mean ± 95% CI: 2.0, 1.9–2.0 vs. 2.5, 2.4–2.6), more educated (mean years of schooling ± 95% CI: 6.7, 6.6–6.8 vs. 3.7, 3.5–3.9), and had a lower BMI (mean± 95% CI: 20.1, 19.9–20.2 vs. 22.5, 22.2–22.7). About 5 percent of the Pakistani women reported having hypertension. Few women in both sites reported having diabetes (Table 1).

**Table 1:** Selected baseline characteristics of study population by study site (N = 4,366).

Characteristics	Study site						Total (N = 4,366)	
	Bangladesh (N = 2,577)		Pakistan (N = 1,789)					
** **	n	%		n	%	n		%
**Mother’s Age**								
≤19 years	557	21.6		141	7.9	698		16
20–29 years	1,714	66.5		1,144	63.9	2,858		65.5
≥30 years	306	11.9		504	28.2	810		18.6
Mean (95% CI)	23.4 (23.3–23.6)			26.6 (26.3–26.8)		24.7 (24.6–24.9)		
**Parity**								
Nulliparous	970	37.6		460	25.7	1,430		32.8
1	666	25.8		410	22.9	1,076		24.6
2–3	727	28.2		572	32	1,299		29.8
>3	214	8.3		347	19.4	561		12.8
Mean (95% CI)	2.0 (1.9–2.0)			2.5 (2.4–2.6)		2.2 (2.1–2.2)		
**Mother’s Education**								
None	155	6.0		887	49.6	1,042		23.9
Primary	924	35.9		324	18.1	1,248		28.6
Secondary and above	1,498	58.1		578	32.3	2,076		47.5
Mean (95% CI)	6.7 (6.6–6.8)			3.7 (3.5–3.9)		5.5 (5.4–5.6)		
**Mother’s BMI**								
<18.5	834	32.4		395	22.1	1,229		28.1
18.5–24.9	1,573	61		889	49.7	2,462		56.4
≥25	170	6.6		505	28.2	675		15.5
Mean (95% CI)	20.1 (19.9–20.2)			22.5 (22.2–22.7)		21.0 (20.9–21.2)		
**Tobacco Use**								
Yes	455	17.7		334	18.7	789		18.1
No	2,122	82.3		1,455	81.3	3,577		81.9
**History of Diabetes**								
Yes	8	0.3		13	0.7	21		0.5
No	2,569	99.7		1,776	99.3	4,345		99.5
**History of Hypertension**								
Yes	6	0.2		88	4.9	94		2.2
No	2,571	99.8		1,701	95.1	4,272		97.8
**History of Stillbirth**								
Yes	191	7.4		110	6.1	301		6.9
No	1,526	59.2		1,341	75	2,867		65.7
Nulliparous	860	33.4		338	18.9	1,198		27.4
**History of Miscarriage**								
Yes	315	12.2		535	29.9	850		19.5
No	1,402	54.4		916	51.2	2,318		53.1
Nulliparous	860	33.4		338	18.9	1,198		27.4
**Husband’s Education**								
None	415	16.1		970	54.2	1,385		31.7
Primary	1,261	48.9		244	13.6	1,505		34.5
Secondary and above	901	35		575	32.1	1,476		33.8
Mean (95% CI)	5.2 (5.0–5.3)			3.7 (3.5–3.9)		4.6 (4.5–4.7)		
**Wealth Quintiles**								
Lowest	499	19.4		304	17	803		18.4
Lower	525	20.4		365	20.4	890		20.4
Middle	528	20.5		358	20	886		20.3
Higher	520	20.2		360	20.1	880		20.2
Highest	505	19.6		402	22.5	907		20.8

**Fig 2:**
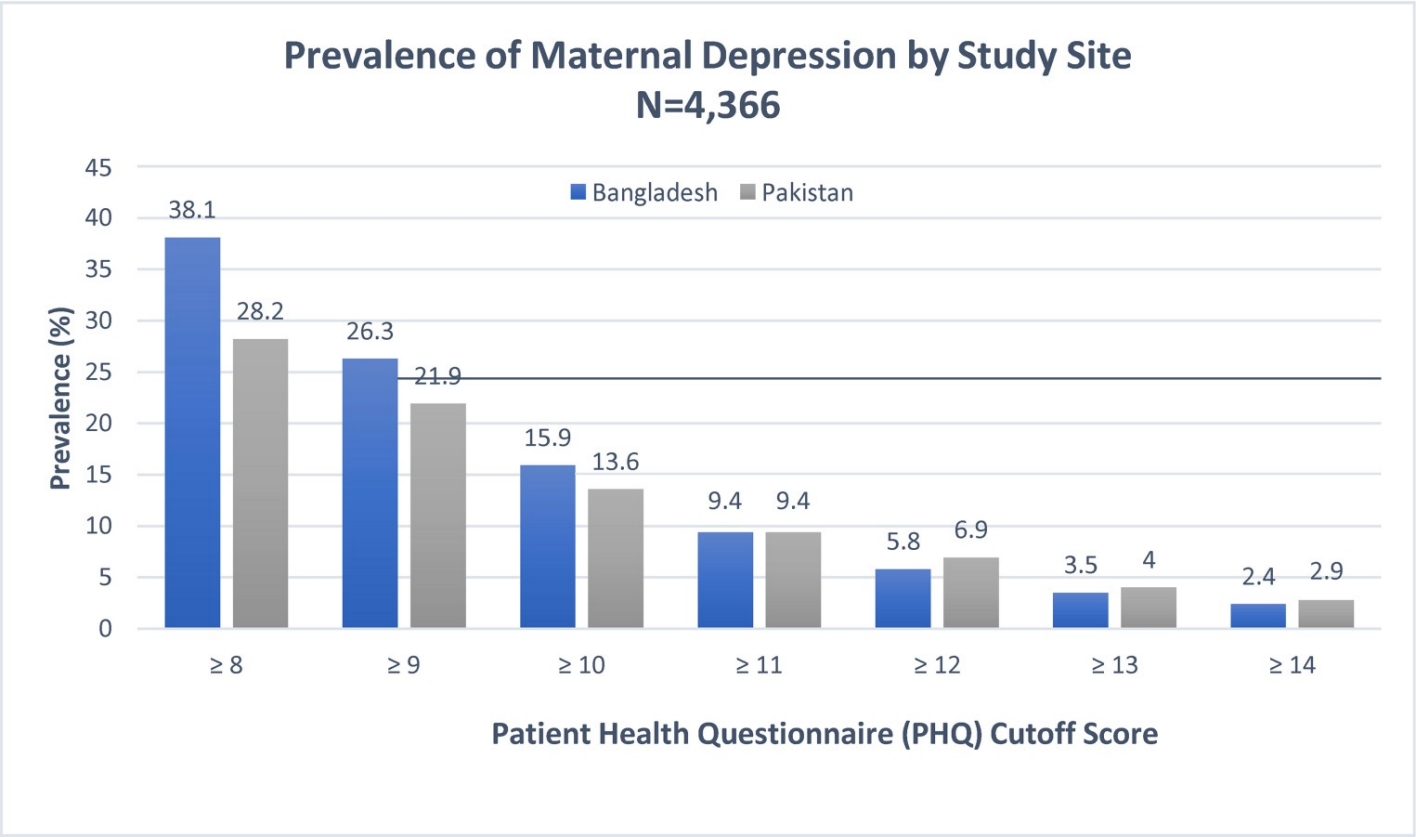
Prevalence of prenatal maternal depression by study site (PHQ-9 cutoff score 8–14).

The prevalence of depressive symptoms during the antenatal period varied between study sites for moderate cutoff scores (PHQ-9 cutoff ≥8–10) with women in Bangladesh more frequently reporting depressive symptoms. However, the prevalence was the same for PHQ-9 **≥**11 and the margins of difference for higher cutoff scores (PHQ-9 cutoff **≥**12 to **≥**14) were about 1.0 percentage point or less between the study sites (Fig 2). Overall, 6.2 percent (N = 272) of the women reported antenatal depression having a score of **≥**12 based on the PHQ-9 questionnaire.

The prevalence of depression was higher among women with higher parity [parity 2–3 (7.1 percent) and parity >3 (8.4 percent)] compared to nulliparous women (5.5 percent) and women with a parity of one (5 percent). Similarly, compared to underweight and normal BMI women, the prevalence of depression was higher among overweight women (8.1 percent) (S1 Table).

In the unadjusted analysis, parity was the only variable significantly associated with women reporting symptoms of depression (Table 2). Compared to women with a parity of one, women with 2–3 children [Risk Ratio (RR), 95% CI: 1.41, 1.02–1.95] and more than 3 children (RR, 95% CI: 1.67, 1.14–2.43) had increased risks of depression (Table 2). In the multivariate analysis, parity and wealth were significantly associated with depression. Women with 2–3 children [Adjusted Risk Ratio (ARR), 95% CI: 1.40, 1.01–1.94] and more than 3 children (ARR, 95% CI: 1.70, 1.15–2.51) were more likely to report depressive symptoms compared to women with a parity of one. Women in the highest wealth quintile were more likely to report depressive symptoms (ARR, 95% CI: 1.54, 1.02–2.34) compared with women in the lowest wealth quintile (Table 2).

**Table 2:** Risk ratios for factors associated with maternal antenatal depressive symptoms (PHQ-9 ≥12).

Variables	Depressive Symptoms (PHQ-9 ≥12)			
	Unadjusted Model		Adjusted Model	
	RR*	95% CI**	ARR***	95% CI
**Mother’s Age**				
20–29 years	Ref	**--**	--	--
≤19 years	0.90	0.64–1.26	--	--
≥30 years	1.12	0.83–1.49	--	--
**Parity**				
1	Ref	--	Ref	--
Nulliparous	1.10	0.79–1.54	1.10	0.79–1.55
2–3	1.41	1.02–1.95	1.40	1.01–1.94
>3	1.67	1.14–2.43	1.70	1.15–2.51
**Mother’s Education**				
None	Ref	--	--	--
Primary	0.82	0.60–1.13	--	--
Secondary and above	0.93	0.70–1.22	--	--
**Mother’s BMI**				
18.5–24.9	Ref	--	Ref	--
<18.5	0.82	0.62–1.09	0.86	0.65–1.14
≥25	1.30	0.97–1.75	1.17	0.85–1.60
**Any Tobacco Use**				
No	Ref	--	--	--
Yes	0.87	0.64–1.20	--	--
**History of Diabetes**				
No	Ref	--	--	--
Yes	1.53	0.41–5.76	--	--
**History of Hypertension**				
No	Ref	--	-	--
Yes	1.73	0.95–3.15	-	-
**History of Stillbirth/Miscarriage**				
No	Ref	--	--	--
Not applicable (Nulliparous)	0.88	0.66–1.16		
Miscarriage	0.95	0.69–1.31	--	--
Stillbirth	0.86	0.53–1.40	--	--
**Husband’s Education**				
None	0.76	0.58–1.02	0.64	0.47–0.89
Primary	Ref	--	Ref	--
Secondary and above	0.83	0.63–1.09	0.74	0.55–0.98
**Wealth Quintiles**				
Lowest	Ref	--	Ref	--
Lower	1.44	0.96–2.15	1.42	0.95–2.12
Middle	1.44	0.97–2.16	1.43	0.95–2.14
Higher	1.36	0.90–2.03	1.36	0.90–2.07
Highest	1.48	0.99–2.20	1.54	1.02–2.34
**Study Site**				
Bangladesh	Ref	--	Ref	--
Pakistan	1.19	0.94–1.50	1.23	0.94–1.61

Note

* Risk Ratio

** 95% Confidence Interval

*** Adjusted Risk Ratio.

In the bivariate analysis examining the factors associated with preterm birth we found that several variables, including antenatal depression (PHQ-9 ≥12), were significantly associated with delivering preterm baby. The incidence of preterm birth was 13.4 percent in the cohort. The risk of delivering preterm was 17.6 percent among women reporting depressive symptoms (PHQ-9 **≥**12) compared to 13.2 percent (N = 539) among women who did not report depressive symptoms (p = 0.036). The other variables that were associated with preterm birth included maternal education, maternal BMI, history of diabetes, history of hypertension, history of stillbirth, history of miscarriage, household wealth, and study site (S2 Table).

The unadjusted Risk Ratio and 95% confidence intervals (RR, 95% CI) of preterm birth among women with antenatal depression was 1.34, 1.02–1.75. The unadjusted RR, 95% CI for other maternal characteristics were as follows: mother’s underweight status (1.34, 1.13–1.59), overweight status (1.34, 1.09–1.65), history of diabetes (2.50, 1.36–4.59), history of hypertension (1.60, 1.08–2.38), history of miscarriage (1.33, 1.09–1.61), history of stillbirth (1.34, 1.02–1.77), and mothers in Pakistan (1.31, 1.13–1.53) (Table 3). Conversely, compared to women in the lowest household wealth quintile, women in the highest wealth quintiles (RR, 95% CI: 0.71, 0.55–0.91) were significantly less likely to deliver preterm (Table 3).

**Table 3:** Risk ratios for factors associated with preterm birth including maternal antenatal depression (PHQ-9 ≥12).

Variables	Preterm Birth			
	Unadjusted Model		Adjusted Model	
	RR*	95% CI**	ARR***	95% CI
**Depression (PHQ≥12)**				
No	Ref	--	Ref	--
Yes	1.34	1.02–1.75	1.34	1.02–1.74
**Mother’s Age**				
20–29 years	Ref	--	Ref	--
≤19 years	1.08	0.88–1.32	1.34	1.04–1.72
≥30 years	1.05	0.86–1.28	0.92	0.73–1.15
**Parity**				
1	Ref	--	Ref	--
Nulliparous	0.88	0.72–1.08	0.74	0.51–1.08
2–3	1.04	0.85–1.27	1.00	0.82–1.23
>3	0.99	0.77–1.28	0.90	0.68–1.21
**Mother’s Education**				
None	Ref	--	--	--
Primary	0.79	0.65–0.97	--	--
Secondary and above	0.76	0.64–0.91	--	--
**Mother’s BMI**				
18.5–24.9	Ref	--	Ref	--
<18.5	1.34	1.13–1.59	1.33	1.12–1.57
≥25	1.34	1.09–1.65	1.30	1.05–1.61
**Any Tobacco Use**				
No	Ref	--	--	--
Yes	1.10	0.91–1.33	--	--
**History of Diabetes**				
No	Ref	--	Ref	--
Yes	2.50	1.36–4.59	2.04	1.10–3.78
**History of Hypertension**				
No	Ref	--	Ref	--
Yes	1.60	1.08–2.38	1.44	0.96–2.16
**History of Stillbirth/Miscarriage**				
No	Ref	--	Ref	--
Not applicable (Nulliparous)	0.94	0.78–1.14	1.13	0.75–1.69
Miscarriage	1.33	1.09–1.61	1.39	1.13–1.70
Stillbirth	1.34	1.02–1.77	1.42	1.07–1.88
**Husband’s Education**				
None	1.06	0.89–1.27	-	
Primary	Ref	--	-	
Secondary and above	0.86	0.72–1.04	-	
**Wealth Quintiles**				
Lowest	Ref	--	Ref	--
Lower	1.02	0.81–1.28	1.00	0.80–1.26
Middle	1.01	0.81–1.27	1.01	0.80–1.27
Higher	0.81	0.64–1.04	0.80	0.62–1.02
Highest	0.71	0.55–0.91	0.70	0.54–0.90
**Study Site**				
Bangladesh	Ref	--	-	
Pakistan	1.31	1.13–1.53	-	

Note

* Risk Ratio

** 95% Confidence Interval

*** Adjusted Risk Ratio.

After adjusting for covariates, antenatal depression remained significantly associated with preterm birth (ARR, 95% CI: 1.34, 1.02–1.74) (Table 3). Additionally, mother’s young age (1.34, 1.04–1.72), maternal underweight (1.33, 1.12–1.57) and overweight status (1.30, 1.05–1.61), history of diabetes (2.04, 1.10–3.78), history of miscarriage (1.39, 1.13–1.70), and history of stillbirth (1.42, 1.07–1.88) were also significantly associated with higher risk of preterm birth (Table 3). Compared to women in the lowest household wealth quintile, women in the highest household quintile were at a significantly lower risk of preterm birth (0.70, 0.54–0.90) (Table 3).

## Discussion

In this population-based cohort study in two South Asian countries–Bangladesh and Pakistan–about six percent of the women reported symptoms of moderate to moderately severe depression (PHQ-9 score ≥12) during the antenatal period. Of the variables we measured, a parity of 2–3 or >3 and highest household wealth status were significantly associated with an increased risk of antenatal depression. The overall incidence of preterm birth was 13.4 percent. The risk of delivering preterm was 17.6 percent among women who reported symptoms of depression compared to 13.2 percent of the women who did not report such symptoms, or only reported symptoms of milder depression (p<0.05). Antenatal depression in women was significantly associated with the risk of preterm birth (ARR, 95% CI: 1.34,1.02–1.74).

The precise prevalence of antenatal depression in LMIC is not well known. A recent systematic review and meta-analysis that included 66 studies from 30 countries estimated that the prevalence of any depression in the antenatal period was 9.5 percent (95% CI: 7.8–11.2; 17 studies; N = 25,592) and moderate to severe depression was 6.3 percent (95% CI 4.8–7.7) [37]. We used a more conservative cut-off of the PHQ-9 score based on evidence that severity is associated with higher risks for adverse outcomes [38] and observed a prevalence similar to the pooled estimate reported by Falah-Hassani et al (2017) [37]. However, the reported prevalence ranged from 1.7 percent to 14.7 percent. The prevalence can vary due to differences in population and methodology including the tool used, type and timing of measurement, the study sample size, adequacy in ascertaining outcomes, and residual confounding [29]. We used PHQ-9 to measure depression. This tool is extensively validated with reports that it provides a reliable and valid measure of depression severity. These characteristics coupled with the succinct nature of the PHQ-9 make it a useful clinical and research tool [33].

The etiology of depression is complex; environment, genetics, and epigenetics may all play important roles [39]. Other risk factors include physical or sexual abuse, low self-esteem, anxiety disorder, borderline personality disorder, post-traumatic stress disorder, chronic diseases such as diabetes, multiple sclerosis, or cancer, alcohol, or drug use, certain prescription medications, family history of depression, and age, gender, race, and geography [40–42]. There is now accumulating evidence from both high-income countries (HIC) and LMIC that older women, women with no or low education, women from poorer households reporting food insecurity, and women experiencing co-morbidities more often experience depression during their pregnancy [43, 44]. These risk factors are also associated with suboptimal birth outcomes including preterm birth[45–48]. In our population, women with higher parity had a significantly higher risk of antenatal depression. There were trends towards a higher risk of depression in older and illiterate women, as well as in women who reported hypertension and diabetes but those associations were not statistically significant.

Compared to women with none to mild depression during the antenatal period, we observed a 33% increased risk of preterm birth among women having moderate to moderately severe depression. A recent systematic review included 14 studies that assessed the risk of preterm birth in women with antenatal depression, eight of them reported a significantly increased risk of preterm birth [49]. The estimated effects in terms of Odds Ratio/Risk Ratio/Hazards Ratio of depression on preterm birth in these studies ranged from 1.07 to 3.73. A population-based study included in this review noted that the risk for preterm birth increases as the severity of depression increases, suggesting a potential dose-response effect [14]. Most of the studies included in the review considered other risk factors and potential confounders of preterm birth such as maternal age, maternal education, parity, history of preterm birth, smoking, substance abuse, and household socio-economic status [49]. The review highlighted that research on antenatal depression and its effect on preterm birth has largely been limited to HIC with very little data from LMIC. Twelve of the 14 studies included in this review were from the USA with one each from Norway and Sweden [49]. Thus, the data we present in this paper is a significant addition to the literature and has substantial public health importance. There are many differences between HIC and LMIC including the varying availability, access, and quality of obstetric care and the variable levels of risk factors known to contribute to both antenatal depression and preterm birth. Data from settings with differing contexts and dissimilar sets of confounding factors may facilitate the understanding of the potential causal relationship between maternal depression and preterm birth.

Our study has several limitations. We used a screening tool, PHQ-9, to measure antenatal depression. Although validated extensively, PHQ-9 is not an ideal tool to measure the severity of depression. Scores above the recommended cut-off values indicate that a respondent is expected to have major depression. Multiple cut-offs have been suggested and used in the literature. As alluded to earlier, recent validation studies against more meticulous assessments suggested that the precision of the measures in predicting major depression improves by using a more conservative cut-off score of 12 rather than 10 [35]. Although we used ultrasound-based gestational age to determine preterm birth, many of the co-variate data we collected were based on interviews and can be subject to recall and other forms of biases. The recall errors may be substantial as well as differential. For example, a woman with depression may be more likely to recall a miscarriage or a stillbirth. Co-morbidities, such as hypertension and diabetes were likely substantially under-ascertained and under-reported in our study. Finally, we did not have data on several potentially important risk factors such as physical or sexual abuse which is also a limitation of the study.

The significance of this study is that we present data for a group that can be easily identified during pregnancy through simple screening and can be prioritized for interventions early in their pregnancy. Several recent studies reported a dose-response relationship where more severe antenatal depression is associated with higher risks for preterm birth and other adverse pregnancy outcomes. Additionally, more severe antenatal depressions may place women at a higher risk for postnatal depression, which has been shown to impact child development by disrupting parenting [50]. Thus, a focus on antenatal depression can contribute to both improvements in women’s health, birth outcomes as well as long-term health and development of children.

The challenge is the lack of mental health services in many LMICs including for women in our study population. According to WHO Mental Health Atlas 2014, about half of the world’s population live in countries where there is less than one psychiatrist for every 100,000 people [51]. Thus, relying solely on specialists to provide health services for people affected by mental health disorders, including depression, will not be feasible, particularly in LMIC. In response to this dearth of mental health care, WHO has developed the Mental Health Gap Action Programme (mhGAP) intending to develop and scale-up care for mental health disorders. The mhGAP approach consists of the identification of interventions that are evidence-based, cost-effective, and feasible in preventing and managing priority mental health conditions and scaling up these interventions in the primary health care programs in LMIC. WHO mhGAP Intervention Guide provides guidelines on the provision of evidence-based management of mental health problems by primary health care (PHC) workers without specialty training in LMIC [52]. Task-sharing and stepped-care approaches have been tested and promoted as strategies to facilitate the integration of mental health care into primary care programs [53]. There is some early evidence suggesting that these strategies are effective in improving outcomes for women with mental health conditions as well as perinatal and child health outcomes in LMIC [54]. There is an urgent need to develop culturally appropriate and feasible approaches to prevent and manage maternal depression and other common mental health problems in diverse settings. This will involve the assessment of existing mental health services available in the general population and identification of country-specific factors relevant to the development and implementation of integrated mental health care at different levels of the health care system, starting with primary care services [53].

In our analyses, we accounted for several known confounders for the association between antenatal depression and preterm birth. The attenuation of the effect of antenatal depression on preterm birth after adjusting for the confounders suggests that depression is likely to occur within an assemblage of other risk factors. Thus, to effectively address the burden of preterm birth, programs will require the development and provision of integrated care addressing multiple risk factors.

## Supporting information

S1 Table Risk factors for maternal antenatal depressive symptoms (PHQ-9 ≥12). (DOCX)

S2 Table Risk factors for preterm birth. (DOCX)
