# Supplementary material for: Burden and risk factors for antenatal depression and its effect on preterm birth in South Asia: A population-based cohort study
Source: PLoS One. 2022 Feb 7;17(2):e0263091. doi: 10.1371/journal.pone.0263091 (PMC8820649; doi:10.1371/journal.pone.0263091)
Supplement: S1 Table — (DOCX) [file pone.0263091.s001.docx]

**S1 Table. Risk factors for maternal antenatal depressive symptoms (PHQ-9 ≥12)**

| **Characteristics** |  | **Depressive Symptoms (PHQ-9 ≥12)**  **(n=272; 6.2%)** | | |
| --- | --- | --- | --- | --- |
|  | **Number of women** | **Number with depressive symptoms** | **%** | **p-value** |
| **Mother’s Age** |  |  |  |  |
| ≤19 years | 698 | 39 | 5.6 |  |
| 20-29 years | 2,858 | 177 | 6.2 |  |
| ≥30 years | 810 | 56 | 6.9 | 0.56 |
| **Parity** |  |  |  |  |
| 0 | 1,430 | 79 | 5.5 |  |
| 1 | 1,076 | 54 | 5.0 |  |
| 2-3 | 1,299 | 92 | 7.1 |  |
| >3 | 561 | 47 | 8.4 | 0.02 |
| **Mother's Education** |  |  |  |  |
| None | 1,042 | 71 | 6.8 |  |
| Primary | 1,248 | 70 | 5.6 |  |
| Secondary and above | 2,076 | 131 | 6.3 | 0.48 |
| **Mother’s BMI** |  |  |  |  |
| <18.5 | 1,229 | 63 | 5.1 |  |
| 18.5-24.9 | 2,462 | 154 | 6.3 |  |
| ≥25 | 675 | 55 | 8.1 | 0.03 |
| **Any Tobacco Use** |  |  |  |  |
| Yes | 789 | 44 | 5.6 |  |
| No | 3,577 | 228 | 6.4 | 0.40 |
| **History of Diabetes** |  |  |  |  |
| Yes | 21 | 2 | 9.5 |  |
| No | 4,345 | 270 | 6.2 | 0.53 |
| **History of Hypertension** |  |  |  |  |
| Yes | 94 | 10 | 10.6 |  |
| No | 4,272 | 262 | 6.1 | 0.07 |
| **History of Stillbirth** |  |  |  |  |
| Yes | 301 | 17 | 5.6 |  |
| No | 2,867 | 186 | 6.5 |  |
| Nulliparous | 1,198 | 69 | 5.8 | 0.62 |
| **History of Miscarriage** |  |  |  |  |
| Yes | 850 | 55 | 6.5 |  |
| No | 2,318 | 148 | 6.4 |  |
| Nulliparous | 1,198 | 69 | 5.8 | 0.73 |
| **Husband’s Education** |  |  |  |  |
| None | 1,385 | 76 | 5.5 |  |
| Primary | 1,505 | 108 | 7.2 |  |
| Secondary and above | 1,476 | 88 | 6 | 0.15 |
| **Wealth Quintiles** |  |  |  |  |
| Lowest | 803 | 37 | 4.6 |  |
| Lower | 890 | 59 | 6.6 |  |
| Middle | 886 | 59 | 6.7 |  |
| Higher | 880 | 55 | 6.3 |  |
| Highest | 907 | 62 | 6.8 | 0.32 |
| **Study Site** |  |  |  |  |
| Bangladesh | 2,577 | 149 | 5.8 |  |
| Pakistan | 1,789 | 123 | 6.9 | 0.14 |
