# Supplementary material for: Burden and risk factors for antenatal depression and its effect on preterm birth in South Asia: A population-based cohort study
Source: PLoS One. 2022 Feb 7;17(2):e0263091. doi: 10.1371/journal.pone.0263091 (PMC8820649; doi:10.1371/journal.pone.0263091)
Supplement: S2 Table — (DOCX) [file pone.0263091.s002.docx]

**S2 Table. Risk factors for preterm birth**

| **Characteristics** |  | **Preterm Birth**  **(n=587; 13.4%)** | | |
| --- | --- | --- | --- | --- |
|  | **Number of women** | **Number** | **%** | **p-value** |
| **Depressive Symptoms (PHQ ≥12)** |  |  |  |  |
| Yes | 272 | 48 | 17.6 |  |
| No | 4,094 | 539 | 13.2 | 0.036 |
| **Mother’s Age** |  |  |  |  |
| ≤19 years | 698 | 99 | 14.2 |  |
| 20-29 years | 2,858 | 376 | 13.2 |  |
| ≥30 years | 810 | 112 | 13.8 | 0.73 |
| **Parity** |  |  |  |  |
| 0 | 1,430 | 174 | 12.2 |  |
| 1 | 1,076 | 149 | 13.8 |  |
| 2-3 | 1,299 | 187 | 14.4 |  |
| >3 | 561 | 77 | 13.7 | 0.36 |
| **Mother's Education** |  |  |  |  |
| None | 1,042 | 169 | 16.2 |  |
| Primary | 1,248 | 161 | 12.9 |  |
| Secondary and above | 2,076 | 257 | 12.4 | 0.01 |
| **Mother’s BMI** |  |  |  |  |
| <18.5 | 1,229 | 193 | 15.7 |  |
| 18.5-24.9 | 2,462 | 288 | 11.7 |  |
| ≥25 | 675 | 106 | 15.7 | 0.001 |
| **Any Tobacco Use** |  |  |  |  |
| Yes | 789 | 115 | 14.6 |  |
| No | 3,577 | 472 | 13.2 | 0.30 |
| **History of Diabetes** |  |  |  |  |
| Yes | 21 | 7 | 33.3 |  |
| No | 4,345 | 580 | 13.3 | 0.01 |
| **History of Hypertension** |  |  |  |  |
| Yes | 94 | 20 | 21.3 |  |
| No | 4,272 | 567 | 13.3 | 0.02 |
| **History of Stillbirth** |  |  |  |  |
| Yes | 301 | 51 | 16.9 |  |
| No | 2,867 | 394 | 13.7 |  |
| Nulliparous | 1,198 | 142 | 11.9 | 0.05 |
| **History of Miscarriage** |  |  |  |  |
| Yes | 850 | 146 | 17.2 |  |
| No | 2,318 | 299 | 12.9 |  |
| Nulliparous | 1,198 | 142 | 11.9 | 0.001 |
| **Husband’s Education** |  |  |  |  |
| None | 1,385 | 203 | 14.7 |  |
| Primary | 1,505 | 208 | 13.8 |  |
| Secondary and above | 1,476 | 176 | 11.9 | 0.09 |
| **Wealth Quintiles** |  |  |  |  |
| Lowest | 803 | 119 | 14.8 |  |
| Lower | 890 | 134 | 15.1 |  |
| Middle | 886 | 133 | 15 |  |
| Higher | 880 | 106 | 12 |  |
| Highest | 907 | 95 | 10.5 | 0.01 |
| **Study Site** |  |  |  |  |
| Bangladesh | 2,577 | 307 | 11.9 |  |
| Pakistan | 1,789 | 280 | 15.7 | 0.00 |
